# Behavioral and Mental Health Problems in Children

**DOI:** 10.3390/children10111820

**Published:** 2023-11-17

**Authors:** Tingzhong Yang, Dan Wu

**Affiliations:** 1 Women’s Hospital, School of Medicine, Zhejiang University, Hangzhou 310058, China; 2 Center for Tobacco Control Research, School of Medicine, Zhejiang University, Hangzhou 310058, China; 3 School of Psychology, Shenzhen University, Shenzhen 518060, China; wudan.tracy@szu.edu.cn; 4 Shenzhen Humanities & Social Sciences Key Research Bases of the Center for Mental Health, Shenzhen University, Shenzhen 518060, China

The stimulus, stress, and behavioral and mental response (SSB) model proposes that various stimuli induce stress and behavioral responses, which may, in turn, lead to health problems [1]. In recent decades, many countries, especially several developing countries, have experienced rapid economic development and social change. This accelerated pace of societal development and transformation invariably escalates social competition, placing considerable mental stress on the youth. Such stress can inadvertently influence their upbringing, potentially leading to significant behavioral and mental health concerns for their offspring. In particular, today’s world is fraught with crisis and uncertainty. The Russian–Ukrainian and Israeli–Hamas wars are a severe crisis affecting the whole world, and they also impact global societal safety and economic stability. In recent years, the COVID-19 pandemic has not only directly led to behavioral and mental health problems in children but also greatly complicated the life and work of young parents. This poses a significant challenge to child raising and development. Children, inherently a vulnerable demographic, are at the epicenter of these crises. Many studies found that behavioral and mental problems occur in children and adolescents globally, especially in developing countries [2,3]. Given the overt nature of these issues, they may affect children’s development, and the consequences can be lasting, potentially correlating with mental disorders persisting into adulthood. Children’s behavioral and mental problems have become a serious social and public health problem. Governments and social health and service organizations around the world must give sufficient attention and take actionable measures to address these problems.

Numerous studies have identified several risk factors related to behavioral and mental problems of young children, including families’ low socioeconomic status, family dysfunction, physical or psychological abuse, traumatic experiences, and parenting problems [4,5,6,7]. However, the majority of prior research only provides a general description of the demographic characteristics and their associations. Such studies cannot propose substantive prevention and control strategies. Additionally, the methodology and outcomes of numerous studies have failed to yield direct evidence aimed at mitigating the long-term persistence and potential exacerbation of such behavioral and mental disturbances into adulthood [8]. Approaches to address this deficiency need to be based on high-quality scientific research to understand the root causes and determine effective strategies to reduce behavioral and mental health problems. In order to achieve these goals, we proposed and published a Special Issue, “Behavioral and Mental Health Problems in Children”, in the journal “*Children*”. In this Special Issue, 13 papers were published, and we are pleased to find that many high-quality papers appeared in the issue. It is truly a global collection of papers, with authors distributed across many countries, including the United States, France, China, Japan, Israel, and Thailand. These studies cover some key issues related to children’s behavioral and psychological problems, such as “Family integration, regulation and children’s health”, “The mealtime behavior problems of children”, “Parents’ daily hassles and parenting approaches to children’s behavior problems”, “Maternal and paternal authoritarian parenting and adolescents’ impostor feelings”, “Family environment and adolescents’ sexual adaptability”, “The influence of parent’s cardiovascular morbidity on child mental health”, “Resilience among left-behind children”, “Sympathy-empathy and the radicalization of young people”, and “the acute stress symptoms in adolescents during the immediate post-earthquake period”. 

These papers provided a comprehensive understanding of the social and behavioral mechanisms leading to behavioral and mental health problems in children. The information obtained from these studies could be helpful in informing health policy; planning prevention strategies; and designing and implementing appropriate, targeted interventions to help reduce behavioral and mental health problems in children. Many researchers have not only realized these challenges but have also put forward reasonable suggestions for policy and prevention applications. 

In this Special Issue, Yang and Zhang posit that children with good health render it possible for a family to develop a concerted parent–child relationship, enabling families to function as unified entities (contribution 1). Such a finding calls for policymakers to consider that children’s health has consequences beyond medical and healthcare relevancy. Indeed, the health of children may be intrinsically linked to the function and vitality of familial structures and, by extension, the harmonious coexistence within a society marked by mutual solidarity and cohesion.

Acar et al. underscore that interventions focusing on stress management may be effective in reducing daily parenting hassles, which may lead to a decrease in practicing negative parenting strategies and, in turn, lead to a reduction in children’s behavior problems (contribution 2). 

Efendi and his collaborators revealed striking results in demonstrating the need for the careful evaluation of adolescents, who, despite the absence of tangible physical injuries, may exhibit signs of acute stress disorder (contribution 3). Moreover, the research emphasizes the importance of attentive consideration of the psychiatric complaints from adolescents who are willing to seek mental health assistance.

Areas for further efforts: A key issue is that some current studies are not designed from any strong theoretical model, particularly those seeking to elucidate the determinants of people’s behavior. This hinders a deeper understanding of children’s behavior and mental problems, signifying an avenue for scholarly enhancement. To the best of our knowledge, some papers still lack an in-depth and comprehensive overview. In terms of the social and behavioral mechanisms of behavioral and mental problems among children, these particular papers did not delve deep into the core of the matter, which is not very helpful for understanding the real world and solving problems [9]. Further studies need to provide root causes with an understanding of the social and behavioral mechanisms leading to behavioral and mental problems among children. 

One objective of this issue is to disseminate knowledge of holistic, multidisciplinary approaches to policy application and to assist in the design and implementation of effective intervention programs. It must be mentioned that some research is not very targeted and is not designed according to the need to reduce behavioral and mental health problems in the current literature. Furthermore, few intervention studies aimed at resolving problems have been implemented. Further research is needed to overcome this shortcoming.
